# Ellagic Acid Potentiates the Inhibitory Effects of Fluconazole Against *Candida albicans*

**DOI:** 10.3390/antibiotics13121174

**Published:** 2024-12-04

**Authors:** Amanda Graziela Gonçalves Mendes, Carmem Duarte Lima Campos, José Lima Pereira-Filho, Aleania Polassa Almeida Pereira, Gabriel Silva Abrantes Reis, Árlon Wendel de Marinho Silva Araújo, Pablo de Matos Monteiro, Flávia Castello Branco Vidal, Silvio Gomes Monteiro, Isabella Fernandes da Silva Figueiredo, Elizabeth Soares Fernandes, Cristina de Andrade Monteiro, Valério Monteiro-Neto

**Affiliations:** 1 Centro de Ciências da Saúde, Universidade Federal do Maranhão, São Luís 65080-805, MA, Brazil; amandagrazielamendes@gmail.com (A.G.G.M.); carmemdlcampos@gmail.com (C.D.L.C.); jlp.filho@outlook.com (J.L.P.-F.); polassa.aleania@gmail.com (A.P.A.P.); gabriel.abrantes@discente.ufma.br (G.S.A.R.); wendell.arlon@discente.ufma.br (Á.W.d.M.S.A.); pm.monteiro@discente.ufma.br (P.d.M.M.); flavia.vidal@ufma.br (F.C.B.V.); silvio.gm@ufma.br (S.G.M.); 2 Instituto de Pesquisa Pelé Pequeno Príncipe, Curitiba 80250-060, PR, Brazil; bellaafigueiredo@hotmail.com (I.F.d.S.F.); elizabeth.fernandes@pelepequenoprincipe.org.br (E.S.F.); 3 Programa de Pós-graduação em Biotecnologia Aplicada à Saúde da Criança e do Adolescente, Faculdades Pequeno Príncipe, Curitiba 80230-020, PR, Brazil; 4 Departamento de Biologia, Instituto Federal do Maranhão, Av. Getúlio Vargas nº 2158/2159, São Luís 65080-805, MA, Brazil; cristinamonteiro@ifma.edu.br

**Keywords:** ellagic acid, fluconazole, anti-*Candida* activity, antibiofilm activity

## Abstract

**Background/Objectives**: Antifungal resistance to azoles, coupled with the increasing prevalence of *Candida albicans* infections, represents a significant public health challenge and has driven the search for new natural compounds that can act as alternatives or adjuvants to the current antifungals. Ellagic acid (EA) has demonstrated antifungal activity; however, its effects are not fully understood. In this study, we investigated the in vitro anti-*Candida* activity of EA and its ability to potentiate the effects of fluconazole (FLZ) on *C. albicans.* **Methods**: The Minimum Inhibitory Concentration (MIC) of EA was determined by broth microdilution and its interaction with FLZ was assessed using a checkerboard assay. Additionally, we examined the effects of EA on yeast-to-hypha transition, inhibition of biofilm formation, time–kill kinetics, hemolytic activity, and cytotoxicity in HeLa ATCC^®^ CCL-2™ cells. **Results**: EA exhibited MIC values ranging from 250 to 2000 µg/mL and showed synergistic and additive interactions with FLZ, resulting in a marked reduction in the MIC values of FLZ (up to 32-fold) and EA (up to 16-fold). In the time–kill assay, the most effective combinations were 4× EA MIC, 2× EA MIC, and FIC EA + FLZ, which showed fungicidal activity. Furthermore, EA did not show hemolytic activity and demonstrated low and dose-dependent cytotoxicity in HeLa cells, with no cytotoxic effects observed in combination with FLZ. EA and the synergistic combination of EA and FLZ interfered with both the yeast-to-hypha transition process in *C. albicans* cells and biofilm formation. In addition to its antifungal efficacy, EA demonstrated a favorable safety profile at the concentrations used. **Conclusions**: This study presents promising results regarding the potential use of EA in combination with FLZ for the treatment of *C. albicans* infections.

## 1. Introduction

Fungal infections represent a serious public health problem as they are associated with increasing rates of morbidity and mortality worldwide. The emergence of microorganisms resistant to conventional antimicrobials has generated great concern among health authorities. In particular, invasive fungal infections stand out because of the scarcity of antifungal classes available for treatment, coupled with the emergence and spread of multidrug-resistant fungal pathogens, which aggravates the scenario and limits effective therapeutic options [1].

The spread of fungal resistance to established antifungal agents and concerns regarding the potential for a global infection crisis have prompted a comprehensive search for solutions to address or mitigate this problem. Fungal resistance constitutes a significant public health concern, as it restricts available treatment options and impedes effective infection control. Consequently, there is an ongoing effort to develop new antifungal compounds and alternative therapeutic approaches to overcome this challenge [1]. In 2022, the World Health Organization (WHO) released its first global priority list of fungal pathogens, highlighting the challenges and hazards to health. This list categorizes pathogenic fungi into three priority levels: critical, high, and medium. Critical fungi include *Aspergillus fumigatus*, *Candida albicans*, *Candida auris*, and *Cryptococcus neoformans*, which pose serious public health risks worldwide [2].

Invasive fungal diseases are frequently caused by *Candida* spp., with *C. albicans* being the most prevalent pathogen. In the United States, it is considered one of the primary sources of bloodstream infections, resulting in a mortality rate of approximately 40%, despite antifungal therapy. In Brazil, the scenario is characterized by challenging diagnosis and treatment procedures, a high fatality rate ranging from 40% to 60%, and substantial hospital expenses [1,3,4,5,6]. *Candida* species, including *C. albicans*, *C. glabrata*, *C. parapsilosis*, *C. tropicalis*, and *C. auris*, are opportunistic microorganisms and therefore coexist harmoniously in most healthy individuals. However, conditions such as a compromised immune system, treatment with broad-spectrum antibacterial agents, and chemotherapeutics make these microorganisms significant agents for both superficial and invasive infections [7,8].

Among the species causing *Candida* spp. infections, *C. albicans* has been the most prevalent, accounting for more than 50% of all cases. However, in recent years, a shift in distribution has been observed, with other non-*albicans Candida* (NAC) species being increasingly identified as human pathogens. In some cases, NAC species have been isolated more frequently than *C. albicans* [8,9]. The pathogenesis of candidiasis depends on the health of the host as well as on the virulence factors expressed by the microorganism, including germ tube formation, adhesion, phenotype switching, biofilm formation, and the production of hydrolytic enzymes [10].

Several factors contribute to the virulence of *C. albicans.* A key feature of this strain is its ability to switch between yeast and hyphal or pseudohyphal forms, enabling adaptation to diverse growth environments. The capacity of an organism to adhere to living and non-living surfaces can lead to biofilm formation, which is particularly problematic for medical devices such as catheters. Additionally, *C. albicans* exhibits exceptional metabolic adaptability, allowing it to quickly adapt to various host environments. This fungus also possesses significant genetic flexibility, facilitating rapid evolutionary adaptation to selective pressures and stressors, including exposure to antifungal drugs [11,12].

The treatment of *C. albicans* infections is limited to three classes of antifungal medications: echinocandins, polyenes, and azoles. This restricted range of options has specific drawbacks that limit its clinical use [13,14]. Echinocandins, for instance, have a narrow antifungal spectrum and are hindered by the need for intravenous administration and their high costs. Polyenes can cause severe kidney damage owing to their nonselective interactions with cholesterol in mammalian cell membranes. Azoles only inhibit the growth of *Candida* species rather than kill them, which can lead to the development and proliferation of drug-resistant strains [11,13,14].

Fluconazole (FLZ), an antifungal drug with minimal side effects, is commonly used as the primary treatment for yeast infections. As an azole antifungal, FLZ acts by impeding the production of ergosterol, a crucial component of the fungal cell membrane. However, the widespread and prolonged use of FLZ, coupled with its fungistatic nature, has resulted in increased resistance and subsequent treatment failure, particularly in recurring infections [10,15,16]. Consequently, there is an urgent need for novel antifungal agents to combat the growing challenges of resistance and severe fungal infections [1].

In this context, the investigation of novel compounds derived from medicinal flora, utilized either alone or in conjunction with conventional antifungal agents such as FLZ, represents a potentially promising avenue. Currently, researchers are exploring combination therapies as alternative strategies for addressing antifungal resistance. These approaches offer the potential for improved efficacy and specificity compared to single-drug treatments while potentially delaying the development of resistant fungal strains [1,17].

Ellagic acid (EA) is a dilactone of hexahydroxydiphenic acid (HHDP), produced through the hydrolysis of ellagitannins, a widely distributed group of plant secondary metabolites. Because of its broad distribution in the plant kingdom, EA has been extensively studied, and several properties have been described for this compound, including antioxidant, anti-inflammatory, antihyperlipidemic, antiviral, antiangiogenic, anticancer, hepatoprotective, cardioprotective, chemopreventive, neuroprotective, antidiabetic, gastroprotective, antidepressant, and anti-apoptotic activities [18,19,20,21].

The antimicrobial potential of EA has also been investigated, primarily against bacteria; however, limited information is available on its antifungal potential and mechanisms [19,21]. Therefore, this study aimed to elucidate the antifungal activity of EA against *Candida albicans*. We investigated the antifungal properties of EA and examined its interactions with varying concentrations of FLZ against resistant and sensitive isolates of *C. albicans*. Additionally, we assessed the activity of EA alone or in combination with FLZ on the yeast–hypha transition and biofilm formation processes of *C. albicans*.

## 2. Results

### 2.1. Minimum Inhibitory Concentration (MIC) and Minimum Fungicidal Concentration (MFC) of EA Against Candida albicans Strains

The MICs of EA and FLZ were determined against 21 clinical isolates and two standard strains of *C. albicans*. EA showed antifungal activity against all tested isolates, with MIC values ranging from 250 to 2000 µg/mL. Among the tested strains, 4.34% had an MIC of 2000 µg/, 30.43% of 1000 µg/mL, 47.82% of 500 µg/mL, and 17.39% of 250 µg/mL (Table 1). Additionally, EA exhibited fungicidal activity against 52.17% of the evaluated strains. However, it was not possible to calculate the MFC activity of EA against 47.82% of the isolates because of the high MFC values of EA, which were higher than 2000 µg/mL, as shown in Table 1.

The MIC of FLZ was determined according to the specific standard for in vitro susceptibility tests for *C. albicans*, in which the sensitivity of the strains evaluated to the FLZ antifungal was determined [22]. Regarding the susceptibility profile, 21.73% were classified as resistant (R), 43.47% as dose-dependently sensitive (D-DS) and 34.78% as sensitive (S). Notably, the vehicle control showed no inhibitory effect on the tested strains.

**Table 1:** Evaluation of the fungal activity of ellagic acid and fluconazole.

Microorganisms	MIC EA (µg/mL)	MFC EA (µg/mL)	MFC/MIC	Activity	MIC FLZ (µg/mL)
Reference strains					
*C. albicans* (ATCC 90028)	500	1000	2	Fungicide	2 (S)
*C. albicans* SC5314 (ATCC MYA-2876)	250	1000	4	Fungicide	8 (S)
Clinical isolates					
*C. albicans* CA 01	1000	2000	2	Fungicide	4 (S)
*C. albicans* CA 02	500	>2000	-	-	8 (S)
*C. albicans* CA 03	1000	>2000	-	-	16 (D-DS)
*C. albicans* CA 04	500	500	1	Fungicide	16 (D-DS)
*C. albicans* CA 05	1000	2000	2	Fungicide	32 (D-DS)
*C. albicans* CA 06	2000	>2000	-	-	8 (S)
*C. albicans* CA 07	500	2000	4	Fungicide	32 (D-DS)
*C. albicans* CA 08	1000	2000	2	Fungicide	64 (R)
*C. albicans* CA 09	500	2000	4	Fungicide	32 (D-DS)
*C. albicans* CA 010	1000	2000	2	Fungicide	64 (R)
*C. albicans* CA 011	500	1000	2	Fungicide	32 (D-DS)
*C. albicans* CA 012	500	2000	4	Fungicide	2 (S)
*C. albicans* CA 013	1000	1000	1	Fungicide	64 (R)
*C. albicans* CA 014	250	>2000	-	-	32 (D-DS)
*C. albicans* CA 015	500	>2000	-	-	64 (R)
*C. albicans* CA 016	500	>2000	-	-	64 (R)
*C. albicans* CA 017	500	>2000	-	-	2 (S)
*C. albicans* CA 018	250	>2000	-	-	<0.5 (S)
*C. albicans* CA 019	250	>2000	-	-	16 (D-DS)
*C. albicans* CA 020	1000	>2000	-	-	16 (D-DS)
*C. albicans* CA 021	500	>2000	-	-	16 (D-DS)

- not calculable, (FLZ) fluconazole, (EA) ellagic acid, (MIC) Minimum Inhibitory Concentration, (MFC) and Minimum Fungicidal Concentration. The MFC/MIC ratio was calculated to determine whether ellagic acid has fungistatic (MFC/MIC > 4) or fungicidal (MFC/MIC ≤ 4) activity [23]. Interpretative values for fluconazole, according to the CLSI [22]: sensitive (S): ≤8 µg/mL; dose-dependently sensitive (D-DS): 16–32 µg/mL; resistant (R): ≥64 µg/mL. All experiments were performed in triplicates in three independent experiments.

### 2.2. In Vitro Interaction of Ellagic Acid with Fluconazole

For the in vitro interaction of EA with FLZ, 11 fungal strains were selected, including those that showed resistance to FLZ (CA 08, CA 010, CA013, CA 015 and CA 016); those that showed dose-dependent sensitivity (D-DS) (CA 04, CA 05 and CA 014); and the sensitive strains (CA ATCC 90028, CA ATCC SC5314 MYA-2876 and CA 02). The initial concentrations used for the in vitro interactions were twice the MIC of EA and FLZ. The combination of EA + FLZ showed synergistic interactions against 54.54% of the strains, additive interactions against 27.27%, and indifference against 18.18%. These results suggested that EA potentiated the antifungal effects of FLZ (Table 2). The fractional inhibitory concentration index (FICI) shown in Table 2 corresponds to the lowest obtained index. Interestingly, even for interactions classified as indifferent, EA reduced the MIC of fluconazole, as shown in the column referring to the fractional inhibitory concentration (FIC) in Table 2.

The in vitro interactions of EA + FLZ with *C. albicans* SC5314 ATCC MYA-2876 and CA 014 showed no additive, synergistic, or antagonistic interactions, resulting in an indifferent interaction. We observed that EA amplified the effect of FLZ with an additive interaction in *C. albicans* ATCC 90028 (IFIC = 0.75) and in various combinations of EA + FLZ analyzed for other strains, as shown in Table 3.

### 2.3. Evaluation of Time–Kill Curve

**Figure 1:**
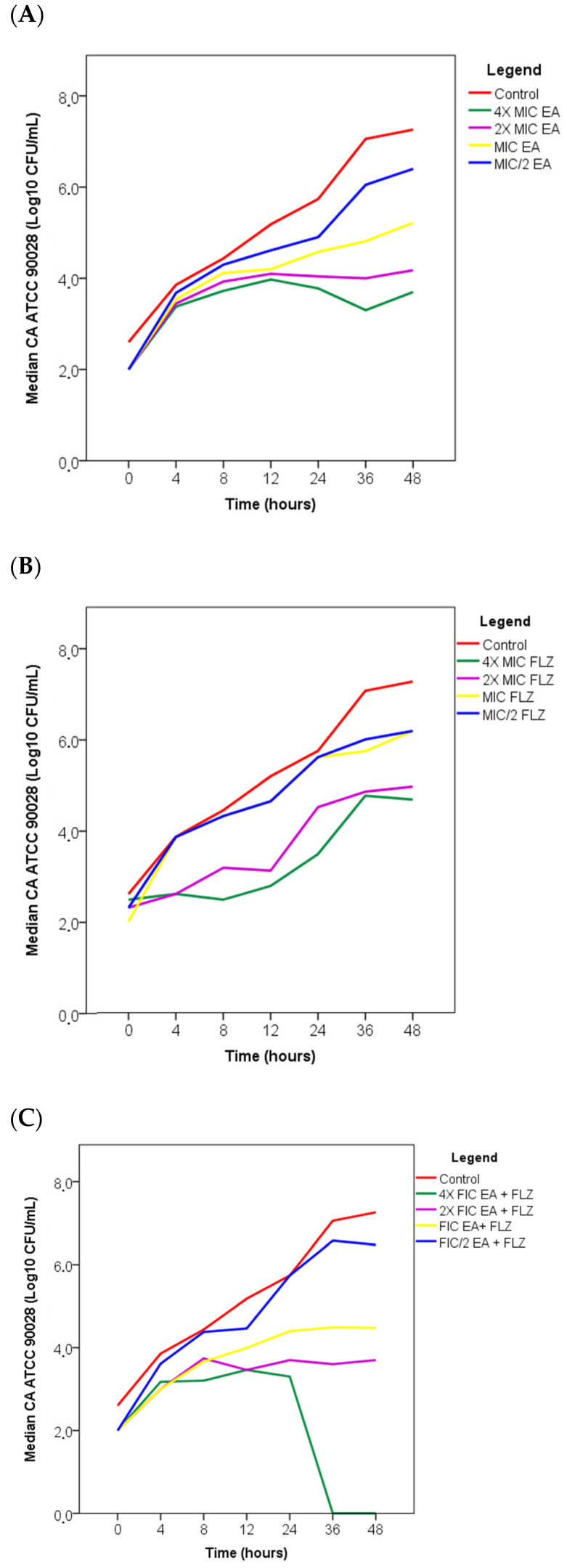
Time-kill curve of *C. albicans* ATCC 90028 under the action of EA (**A**) and FLZ (**B**) and their combination (FIC EA + FLZ) (**C**) showing the medians. The Shapiro–Wilk test was used to assess the normality of the sample. Significant differences between groups were determined by Kruskal–Wallis analysis followed by Dunn’s test with *p* > 0.05 considered significant. Legend: FIC = fractional inhibitory combination, MIC = minimal inhibitory concentration, EA = ellagic acid, FLZ = fluconazole.

**Figure 2:**
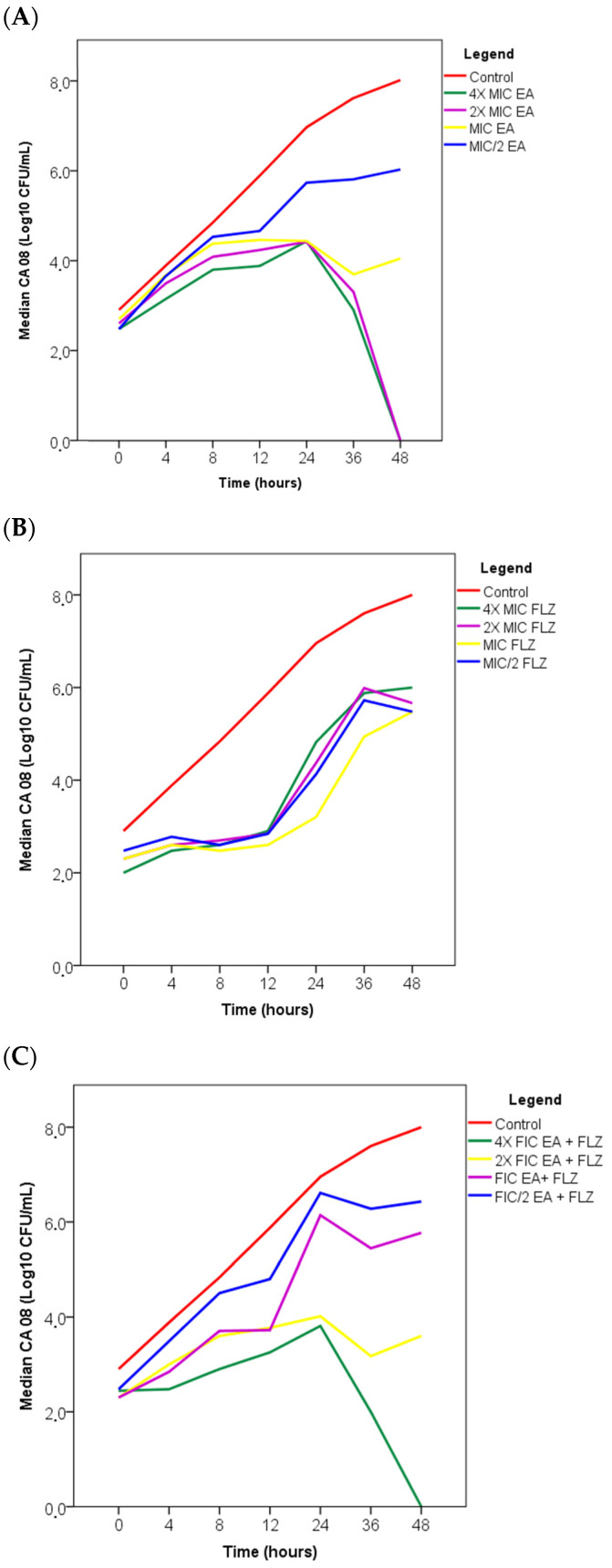
Time-kill curve of *C. albicans* CA 08 under the action of EA (**A**), FLZ (**B**) and in combination (FIC EA + FLZ) (**C**), at different concentrations and control. The Shapiro–Wilk test was applied to analyze normality. Significant differences between groups were determined by Kruskal-Walli’s analysis followed by Dunn’s test, presenting the medians test with (*p* > 0.05) considered significant. Legend: FIC = fractional inhibitory combinations, MIC = minimal inhibitory concentration, EA = ellagic acid, FLZ = fluconazole.

To evaluate the antifungal activity of EA, FLZ, and EA + FLZ at different time intervals, a time–kill curve assay was performed for 0–48 h against *C. albicans* at various concentrations. Two fungal strains were used to evaluate the time–kill curve: a standard strain of *C. albicans* (ATCC 90028), which showed additive interactions (Table 2) and sensitivity to FLZ (MIC = 2 μg/mL) (Table 1), and a clinical sputum isolate (Table 4) of *C. albicans* (CA08), which was resistant to FLZ (MIC = 64 μg/mL) (Table 1) and showed synergistic interactions and the lowest FICI (0.09) among the fungi tested (Table 2). Figure 1 and Figure 2 illustrate the effects of treatment with the compounds, both alone and in combination, on the growth of *C. albicans* ATCC 90028 and clinical isolate CA08, respectively, at different time intervals (0, 4, 8, 12, 24, 36, and 48 h) compared to the untreated control (control).

As shown Figure 1A, the groups that received EA concentrations 4× and 2× after 48 h of incubation showed a reduction in the number of viable cells of *C. albicans* ATCC 90028, with growth of 3.69 and 4.17 Log10 CFU/mL, respectively, compared to the control, which showed 7.26 Log10 CFU/mL, indicating a potential fungicidal effect of the compound. In contrast, at the MIC and MIC/2 concentrations of EA, no reduction in the number of cells by ≥3 Log10 CFU/mL was observed, suggesting fungistatic activity (Figure 1A).

Figure 1B shows that FLZ treatment did not result in significant reductions in the number of *C. albicans* ATCC 90028 cells by ≥3 Log10 UFC/mL, confirming the fungistatic action of FLZ.

The fractional inhibitory combinations (FICs) of EA + FLZ (Figure 1C) used were shown to significantly reduce fungal cells in the FIC 4× (no growth) and 2× EA + FLZ (3.69 Log10 CFU/mL) after 48 h compared to the control which showed a 7.26 Log10 CFU/mL, suggesting a fungicidal effect on *C. albicans* ATCC 90028. At FIC and FIC/2 EA + FLZ concentrations, there was no significant reduction in cells by ≥3 Log10 CFU/mL, indicating fungistatic activity.

Time–kill tests with the clinical isolate *C. albicans* CA08 (Figure 2A) revealed that treatments with 4× and 2× MIC of EA resulted in no growth after 48 h, with a 99.99% reduction in cells, suggesting a fungicidal effect. The untreated control group showed growth of 8 Log10 CFU/mL. The AE MIC and MIC/2 treatments showed no reduction in the number of viable fungal cells by ≥3 Log10 UFC/mL, suggesting fungistatic activity.

After the 48 h incubation period, the FLZ treatments were unable to reduce the number of viable fungal cells to ≥3 Log10 UFC/mL, with fungistatic activity (Figure 2B). On the other hand, the strains treated with 4× FIC EA + FLZ (Figure 2C) showed no growth after 48 h; 2× FIC EA + FLZ showed logarithmic growth of 3,6 Log10 UFC/mL, compared to the control of 8 Log10 UFC/mL, which suggests a fungicidal effect. The FIC and FIC/2 EA + FLZ treatments were unable to reduce the number of viable cells with logarithmic values of 5.77 and 6.46 Log10 UFC/mL, respectively, concerning the control of 8 log10 UFC/mL, which suggests fungistatic activity.

### 2.4. Inhibition of Hyphae and/or Pseudohyphae Formation

We evaluated the effects of EA, FLZ, and their combinations on the formation of hyphae and/or pseudohyphae in four selected strains: a standard reference strain, *C. albicans* ATCC 90028, sensitive to FLZ (Table 1) and with additive interaction (Table 2), and three clinical isolates, *C. albicans* CA 08 from sputum, *C. albicans* CA 010, and CA 013 isolated from urine (Table 4), resistant to FLZ (Table 1) and with the lowest ICIF among the other fungi (Table 2).

**Figure 3:**
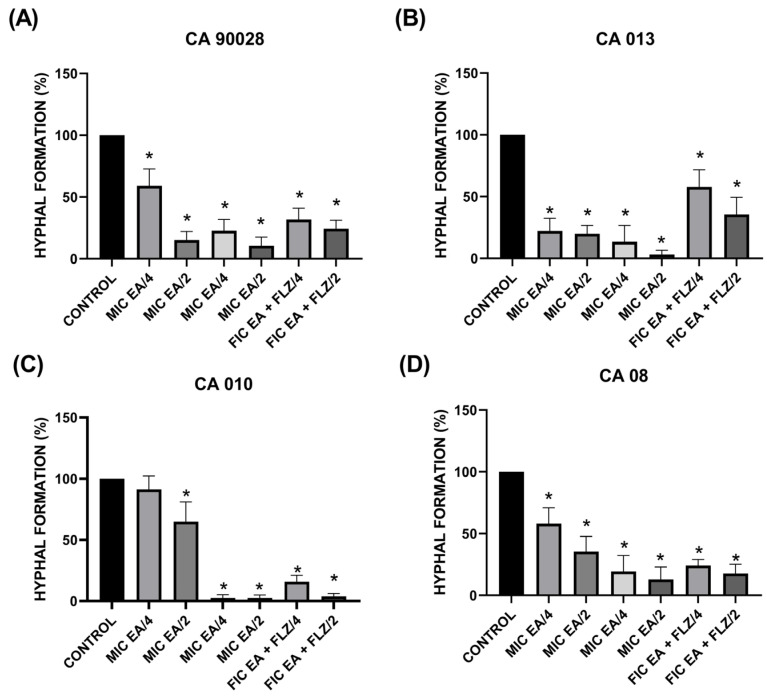
*C. albicans* hyphae formation. Clinical isolates: (**A**) CA 90028; (**B**) CA 013; (**C**) CA 010; (**D**) CA08. The Shapiro–Wilk normality test was applied. The results are presented as mean and standard deviation; significant differences between groups were determined by one-way analysis of variance (ANOVA) followed by Tukey’s multiple comparison where the value of *p* < 0.05 (*) was considered significant.

Figure 3 shows the percentage of *C. albicans* hyphae formation under the action of EA, FLZ, and their combination (FIC EA + FLZ) at sub-inhibitory concentrations. The percentage of hyphae formation per 100 cells was calculated and compared with that of the untreated control (control). We observed that, with the exception of the MIC EA/4 group in the treatment of strain CA 08 (Figure 3C), all treated groups showed a significant reduction (*p* < 0.05) in the hyphae formation rate, compared to the untreated control (Figure 3).

### 2.5. Antibiofilm Activity

**Figure 4:**
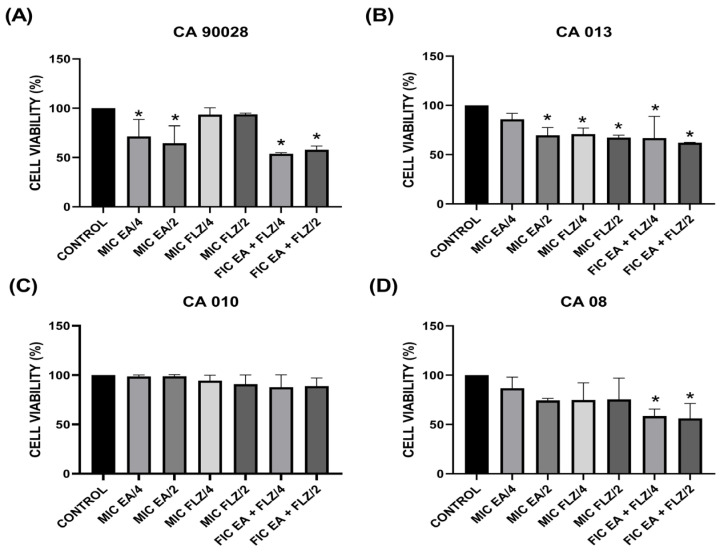
Cell viability of young *C. albicans* biofilms. Clinical isolates: (**A**) CA 90028; (**B**) CA 013; (**C**) CA 010; (**D**) CA08. The Shapiro–Wilk normality test was applied. Results are presented as mean and standard deviation; significant differences between groups were determined by one-way analysis of variance (ANOVA) followed by Tukey’s multiple comparison where the value of *p* < 0.05 (*) was significant.

For the biofilm experiments, four yeasts selected for the hyphae formation interference test were used. Figure 4 shows the percentage cell viability of the young biofilm of the different treated strains compared to the untreated control group (control).

Figure 4A,B show that the groups treated with sub-inhibitory concentrations of EA alone reduced the formation of young biofilms of *C. albicans* ATCC 90028 and CA013. The CA013 FLZ/2 and FLZ/4 groups showed significant reductions (*p* < 0.05) (Figure 4B).

The sub-inhibitory concentrations of the FIC EA + FLZ combination proved to be effective in treating young biofilms of *C. albicans* ATCC 90028, CA 08, and CA 013 (Figure 4A,B,D) and ineffective in treating biofilms of CA 010 (Figure 4C) compared with the untreated control.

**Figure 5:**
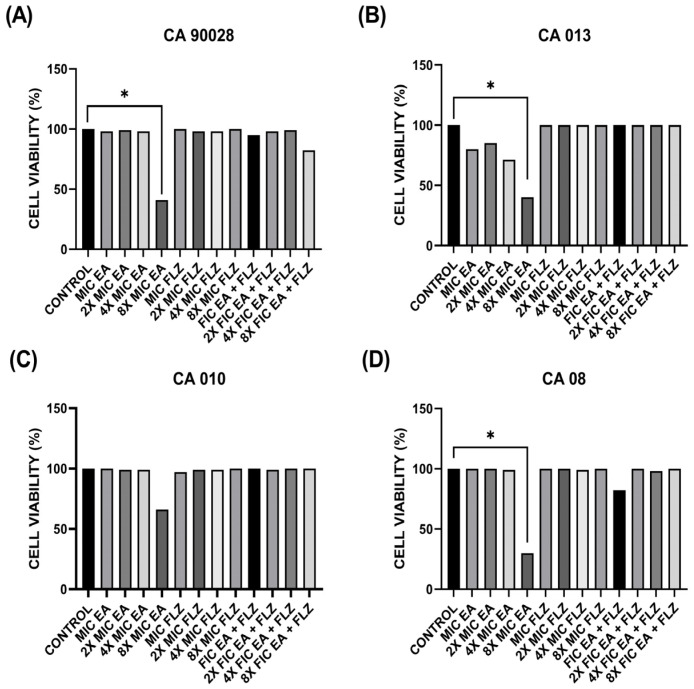
Cell viability of mature *C. albicans* biofilms. Clinical isolates: (**A**) CA 90028; (**B**) CA 013; (**C**) CA 010; (**D**) CA08. The Shapiro–Wilk normality test was applied. Significant differences between groups were determined by Kruskal–Wallis analysis of variance followed by Dunn’s test, where the value of *p* < 0.05 (*) was significant.

In the interference tests on the mature biofilm, four yeast strains used in the young biofilm were tested. Figure 5 shows the percentage of cell viability of the mature biofilms of the different strains under the action of the treatment compared with the untreated growth control (control).

Only the EA 8× MIC treatment group showed significant activity (*p* < 0.05) against the mature biofilms of *C. albicans* ATCC 90028, CA08, and CA013, as shown in Figure 5A,B,D. None of the treatments used were able to inhibit or eradicate the CA 010 biofilm, as shown in Figure 5C.

### 2.6. Hemolytic Activity of Ellagic Acid

**Figure 6:**
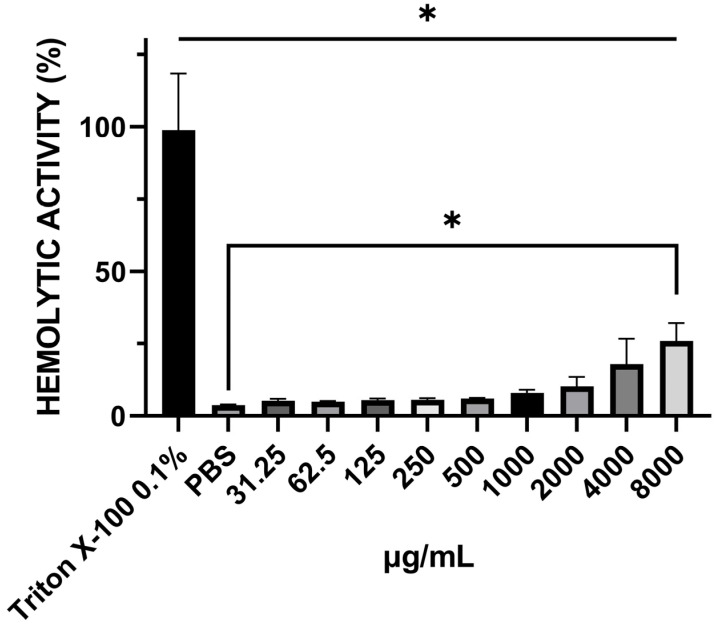
Hemolytic activity. The Shapiro–Wilk normality test was applied. The test results are presented with mean and standard deviation; significant differences between specific groups were determined by one-way analysis of variance (ANOVA) followed by Tukey’s multiple comparison, where the value of (* *p* < 0.05) was significant.

This assay aimed to assess whether EA could induce hemolysis and examine its potential toxic effects. The percentage of hemolytic activity was obtained by comparing the data from the groups treated with EA at different concentrations, as well as its vehicle (78–2500 µg/mL NaOH), compared to the 0.1% Triton X-100 solution (positive control, Sigma-Aldrich, Saint Louis, MO, USA). Different concentrations of EA and vehicle showed no hemolytic activity compared to the positive control (0.1% Triton X-100); even at the highest concentration of EA (8.000 μg/mL), we observed a percentage of hemolysis of only 24.53% (Figure 6).

### 2.7. Cytotoxic Activity

**Figure 7:**
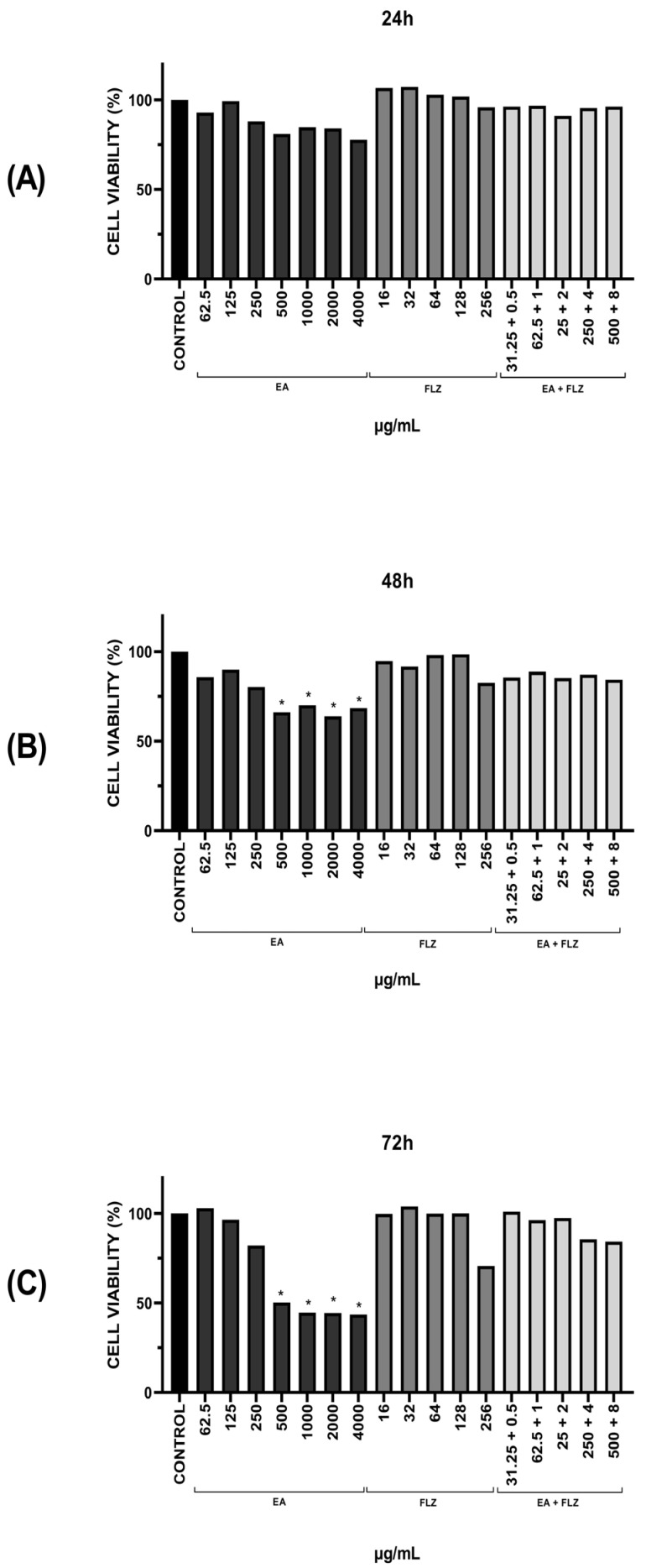
Cytotoxicity assay in HeLa. Cell viability was evaluated after (**A**) 24 h, (**B**) 48 h, and (**C**) 72 h. The Shapiro–Wilk normality test was applied. Significant differences between groups were determined by Kruskal–Wallis analysis followed by Dunn’s multiple comparisons test, where the value of (* *p* < 0.05) was significant. EA = ellagic acid; FLZ = fluconazole.

The cytotoxic activities of EA, FLZ, and EA + FLZ were evaluated against a human cervical carcinoma cell line (HeLa-ATCC^®^ CCL-2™, ATCC, Manassas, VA, USA) using the MTT cell viability assay, which can be converted into formazan, indicative of cell viability [25]. No cytotoxic effects were observed for the combinations tested at the different incubation periods evaluated (24, 48, and 72 h) (Figure 7).

Figure 7A shows that in all treatments, even at the highest concentrations tested, no significant cytotoxic effects were observed in HeLa cells (ATCC^®^ CCL-2™) after 24 h of incubation, indicating high cell viability.

After 48 h (Figure 7C), significant reductions in cell viability were observed only at EA concentrations of 500–4000 μg/mL and in the vehicle, with no evidence of effects in the FLZ assays (Figure 7B). Figure 7C shows cytotoxic effects after 72 h of EA treatment at concentrations of 500 to 4000 μg/mL. However, EA concentrations below 500 μg/mL did not significantly reduce cell viability after 72 h.

## 3. Discussion

The antimicrobial activity of EA has been previously demonstrated [19,26,27,28,29,30,31]. Here, analysis of the in vitro antifungal activity of EA and its combination with FLZ showed its efficacy against *C. albicans*, corroborating the findings in the literature. We obtained a better understanding of the antifungal properties of high-purity EA and its effects on *C. albicans*. Although some studies have already demonstrated the antifungal activity of EA [32,33], this study showed that EA could exert activity against all the yeasts analyzed, including ATCC strains and clinical isolates. The MIC values ranged from 250 to 2000 μg/mL. This demonstrates the antifungal efficacy of EA against a group of 23 *C. albicans* isolates. Furthermore, the effect of EA on clinical *C. albicans* isolates of different origins was also demonstrated.

In a previous study, Silva Junior et al. (2010) analyzed the antifungal activity of EA obtained from *Lafoensia pacari* against yeasts of the genus *Candida* using broth microdilution, which resulted in MIC values ranging from 125 to 1000 μg/mL [33]. Lower MIC values, ranging from 25 μg/mL to 75 μg/mL, were also observed against *Candida* spp. in a study by Li et al. (2015) [26]. Possamai Rossatto et al. (2021) analyzed plants containing EA in their composition in relation to their antifungal and antiviral properties and verified activity against *C. auris* and *C. albicans* [34].

The varied MIC values identified in our study, in both planktonic and biofilm forms, may be linked to the high genomic plasticity exhibited by *Candida* spp., which can lead to changes in the genome and gene expression between different species or between strains of the same species [35,36].

Furthermore, the existence of various virulence factors and strategies for resistance, adaptation to environmental conditions, and reactions to multiple stresses can affect the sensitivity of various *Candida* species and strains to a given substance [35,36]. These variations could also be linked to the different growth conditions, purity of the compounds, and origin of the strains [34].

Our results showed that EA and its combination with FLZ, the antifungal of choice for the treatment of candidiasis, had synergistic or additive interactions with most of the strains used. These data suggested that EA potentiated the antifungal effects of FLZ. The search for associations with antifungals is relevant because of the increase in *Candida* isolates resistant to conventional antifungals, such as FLZ. With the low availability of antifungal drugs, it is necessary to search for new antimicrobials as well as substances with synergistic action, which can improve antifungal activity [37,38,39].

In vitro studies on the interactions between EA and azoles, such as FLZ, against *Candida* are still scarce. It is crucial to examine the combined action of EA and FLZ, which acted synergistically against 54.54% of the strains and additively against 27.27% of the strains. Even in non-synergistic scenarios, such as interactions considered indifferent, the associations reduced the MIC of FLZ. This indicates that the combination of EA and FLZ can decrease the concentration of both by interfering with the in vitro growth of strains, especially the resistant ones, thereby intensifying the antifungal action of the drug. The clinical importance of these findings is relevant because a combination of antimicrobial agents provides a lower inhibitory concentration. In a study by Possamai Rossato et al. (2020) [34], interactions between EA and antifungals were also found; however, unlike this study, only indifferent interactions were observed.

These results suggest that EA could be useful as an adjuvant to FLZ therapy, given that reduced doses of this commercial antifungal could be used in conjunction with EA to treat infections caused by *C. albicans*, with lower associated concentrations of the two, thereby reducing toxic effects and resistance. However, this hypothesis requires further investigation using in vivo tests.

In this study, EA, alone or in combination with FLZ, in sub-inhibitory concentrations, inhibited the transition from yeast to hyphal morphology. This finding suggests that EA and/or its combinations are effective in combating virulence factors presented by fungi. Morphological transition in *C. albicans* is one of the main factors related to its virulence, invasion, and pathogenesis in the host [40].

Regarding *C. albicans* biofilms, EA showed antibiofilm activity alone and when combined with FLZ, interfering with the cell viability of young biofilms of three strains, but not with the clinical isolate CA 010. Studies have indicated that *C. albicans* demonstrates remarkable genetic adaptability, facilitating its rapid evolution and adjustment to various selective pressures, including the presence of antifungal compounds [11,12]. This characteristic may be reflected in the varied drug susceptibility profiles observed among different strains. However, inhibition of biofilm formation is especially important in the management of non-planktonic cells, which presents significant challenges in treatment, with biofilm formation being an important process for the lack of action of antifungals [41,42].

In addition, EA did not show hemolytic activity at the concentrations tested in sheep blood, and research on the hemolytic activity of isolated EA is limited. However, in a previous study [34], a hemolysis test was performed for different concentrations of EA, which showed no hemolytic activity. These results corroborate those reported in the present study.

The cytotoxicity of EA, FLZ, EA + FLZ, and the controls was tested using HeLa cells (ATCC^®^ CCL-2™). Although some of the treatments showed cytotoxicity within 48–72 h, most were considered to have low cytotoxicity for this type of cell between 24 and 72 h. However, other studies have indicated that the cytotoxic potential mediated by EA occurs in a dose-dependent manner, with results showing that EA inhibits the growth of HeLa cells [43]. EA extracted from pomegranate peel inhibits tumor proliferation by regulating the expression of IGBPF7 [44]. Pani et al. (2021) [45] verified the cytotoxic effect of EA (25 μmol/L) on HeLa cells for 96 h, and the compound induced cytotoxicity with an IC_50_ value of 15.84 μM.

As highlighted in this study, the absence of cytotoxicity is a highly valued attribute of therapeutic compounds, as it increases safety, reduces adverse effects and expands treatment options. Here, the combinatorial concentrations of EA and FLZ proved to be effective and safe for possible use. However, we also noted that dose-dependent cytotoxicity was observed over time in treatments with EA alone, indicating that the toxic effect of this agent on cells intensifies as the dose increases and time progresses. However, we also emphasize that most of the MIC values obtained for EA have low or no cytotoxicity, which means that, at reduced doses, the agent can be used in a way that is not harmful or even harmless to cells.

Some limitations can be highlighted in this study, including the evaluation of the activity of EA and its combinations with FLZ against other species of *Candida*, as well as the need for animal studies to further evaluate efficacy and safety. However, considering all the results, EA appears to be a possible candidate for future therapeutic formulations.

## 4. Materials and Methods

Clinical isolates were obtained under ethical precepts, according to the opinion of 813.402 CEP/UNICEUMA. EA was obtained commercially from Sigma-Aldrich, USA^®^, with a purity level ≥ 95% (HPLC); solubilized in 1 molar of sodium hydroxide in phosphate buffer pH 7.5–8.0, according to the manufacturer’s recommendations; taken to the sonicator [22,38]; and then filtered through a 0.22 µm micropore membrane. The antifungal (FLZ) was obtained from Sigma-Aldrich (Saint Louis, MO, USA), dissolved according to the manufacturer’s recommendations, and stored at −20 °C.

### 4.1. Strains and Inoculum Standardization

*C. albicans* species were used for the in vitro analysis. Fungi from the American Type Culture Collection and clinical isolates from the culture collections of the Basic and Applied Microbiology Laboratory–LMBA of the Federal University of Maranhão—UFMA/Brazil and the Microbiology, Bioprospecting and Biotechnology Research Laboratory—LPBIOTEC of the Federal Institute of Maranhão—IFMA/Brazil, all identified by VITEK II—BIOMÈRIEUX (Table 4). To standardize the inoculum, the strains were suspended in phosphate-buffered saline (PBS) to a turbidity concentration of approximately 1 × 10^6^ CFU/mL (Colony-Forming Units per milliliter; equivalent to McFarland scale 0.5). The optical density (0.08–0.1) of the suspensions was measured by spectrophotometry (λ: 530 nm), and they were subsequently diluted 1:20 and 1:50 in RPMI 1640/MOPS to obtain a final inoculum of 1 × 10^3^ CFU/mL on the plate [22].

### 4.2. Determination of Minimum Inhibitory Concentration (MIC)

The antifungal activity was determined using the broth microdilution method. The assays were carried out in a sterile 96-well flat-bottom microplate (Global Trade) with RPMI-1640/MOPS culture medium (Sigma-Aldrich). The final inoculum was 1 × 10^3^ CFU/mL. EA was used at concentrations of 31.5–2000 µg/mL. FLZ was used at concentrations ranging from 64 to 0.5 µg/mL. A growth control, sterile medium, and vehicle were used as controls. The plates were incubated at 37 °C for 24–48 h. MIC was defined as the lowest concentration of the agent capable of inhibiting yeast growth, detected by the lack of visual turbidity [22], and resazurin was used to assess cell viability.

### 4.3. Minimum Fungicide Concentration (MFC)

After determining the MIC, 10 μL aliquots from the wells corresponding to the MIC and higher concentrations were incubated on Sabouraud Dextrose Agar (SDA) plates for 24 h at 37 °C. The concentration without fungal growth was defined as MFC. The MFC/MIC ratio was calculated to determine whether EA had fungistatic (MFC/MIC > 4) or fungicidal (MFC/MIC ≤ 4) activity [23].

### 4.4. In Vitro Interaction

The results of combining EA with FLZ were evaluated using broth microdilution and checkerboard methods [46]. The assays were carried out in sterile 96-well microplates with RPMI-1640/MOPS culture medium (Sigma-Aldrich). The inoculum concentration in the plate was 1 × 10^3^ CFU/mL, concentrations 2× higher than the MIC of the agents used, and the inoculum was incubated at 37 °C for 24–48 h. The results were evaluated by IFIC (Inhibitory Fractional Concentration Index) analysis. The MIC of FLZ and EA in combination was defined as the lowest concentration of the combined agents that inhibited fungal growth. The interaction between EA and FLZ was expressed as the sum of the fractional inhibitory concentrations (FICs) of each agent. The FIC of each agent was calculated as the MIC of the agent in combination divided by the MIC of the agent alone. The assays were performed in triplicates in three independent experiments. Resazurin was used to assess cell viability. The calculated IFIC was as follows:

$$\frac{MIC\;EA\;combined\;}{MIC\;EA\;alone}+\frac{MIC\;FLZ\;combined}{MIC\;FLZ\;alone}$$

IFIC was interpreted as previously described [24]:

- Synergistic: IFIC ≤ 0.5;
- Additive: 0.5 < IFIC ≤ 1.0;
- Indifferent or no interaction: 1.0 < IFIC ≤ 4.0;
- Antagonist: IFIC > 4.0;

### 4.5. Time–Kill Curve

The death curve of EA with FLZ in relation to microbial growth over time was determined as described previously [46,47]. Standardized inocula at a final concentration equivalent to 1 × 10^3^ CFU/mL were deposited in 96 microdilution wells with varying concentrations (4× MIC EA, 2× MIC EA, MIC EA, MIC EA/2, 4× MIC FLZ, 2× MIC FLZ, MIC FLZ, MIC FLZ/2, 4× MIC EA + FLZ, 2× MIC EA + FLZ, MIC EA + FLZ, MIC EA + FLZ/2). The microplates were then incubated at 37 °C for 48 h. During this period and at different time intervals, starting with the moment of inoculation (time zero), followed by 4 h, 8 h, 12 h, 24 h, 36 h and 48 h, aliquots of 100 μL were removed from each well, serially diluted in 900 μL of sterile PBS, and seeded in ASD medium. The Petri dishes were incubated at 37 °C for 24 h and the colonies were counted. A mortality curve was constructed as a function of the number of log 10 CFU/mL recovered over time, and the curves were constructed and compared to the untreated fungal growth control, where the death of ≥3 log 10 CFU/mL (99.9%) cells was considered fungicidal and <3 Log10 CFU/mL (<99.9%) fungistatic, as previously described [47,48].

### 4.6. Inhibition of Yeast–Hypha Stage Transition

The effect of EA on the yeast–hyphae transition was determined according to a previously described methodology with modifications [49]. The inocula standardized at 1 × 10^6^ CFU/mL were placed in 24-well plates containing sub-inhibitory concentrations of MIC (EA/2, EA/4, FLZ/2, FLZ/4) and FIC (EA + FLZ/2 and EA + FLZ/4) in a 1:1 ratio (RPMI-1640/MOPS culture medium) with 10% fetal bovine serum, with a final volume of 1 mL, and incubated at 37 °C for 4 h with agitation at 100 rpm. Subsequently, 100 cells (hyphae and yeasts) were counted on a glass slide, and the percentage of filamentation was calculated (%) as previously described [49]. The percentage of filamentation was determined as follows: No. of hyphae in 100 cells of the untreated growth control = 100%/No. of hyphae in 100 cells in the treatment group.

### 4.7. Action of AE and FLZ and Their Combinations in Biofilm Formation

#### 4.7.1. Young Biofilm

The interference of EA in the formation of young biofilms was performed as described previously [50] in 96-well flat-bottomed microplates. Biofilm adhesion was performed using 200 µL of standardized inoculum solution (1 × 10^6^ CFU/mL) incubated for 90 min at 37 °C. The wells were then washed three times with PBS to remove non-adherent cells, and 200 µL of the treatment diluted in RPMI-1640/MOPS (1:1) was added at sub-inhibitory MIC concentrations (EA/2, EA/4, FLZ/2, and FLZ/4) and FIC (EA + FLZ/2 and EA + FLZ/4). For growth and sterility controls, the plates were incubated at 37 °C for 24 h. Next, the wells were washed three times with PBS and stained with MTT (3-4,5-dimethyl-thiazol-2-yl-2,5-diphenyltetrazolium bromide) [51], and the absorbance was measured using a UV–visible spectrophotometer (λ: 530 nm).

#### 4.7.2. Mature Biofilm

Analysis of the effect of EA on mature biofilms followed the same procedure adopted for the interference of biofilm formation until adhesion, as previously described [50,51]. After washing, 200 µL of RPMI-1640/MOPS medium was added, and the microplates were incubated for 24 h at 37 °C. Then, the microplates were washed three times with PBS and culture medium with treatment (1:1) at varying concentrations (8× EA, 4× EA, 2× EA, MIC EA, 8× FLZ, 4× FLZ, 2× FLZ, MIC FLZ) of FIC (8× EA + FLZ, 4× EA + FLZ, 2× EA + FLZ). The plates were incubated for another 24 h at 37 °C and then washed three times with PBS before assessing the metabolic activity using an MTT assay. Absorbance was measured using a UV–visible spectrophotometer (λ = 530 nm) [51].

### 4.8. Hemolytic Activity

The methodology used was from [52,53,54]. Sheep blood samples were provided by the Central Bioterium of UFMA/Brazil and were collected in tubes containing EDTA-K2 as an anticoagulant. Sheep blood (10 mL) was centrifuged at 1.500 rpm for 5 min at 20 °C to avoid coagulation, and the resulting plasma fraction was removed from the samples. The pellets were washed with an equal volume (10 mL) of PBS and mixed by inversion. The centrifugation and washing steps were repeated thrice, after which a final volume diluted to 1:10 in PBS was added to obtain a concentration of ~5 × 10^8^ erythrocytes/mL. The assay was performed in a 96-well microplate, and the concentrations of EA (31.25–8000 µg/mL) and vehicle (78–2500 µg/mL NaOH) were tested in serial two-fold dilutions in PBS, and 0.1% Triton X-100 was used as a hemolysis control. Negative control was performed with PBS. One hundred microliters of each solution, EA and diluted NaOH solutions, was added to the wells, followed by 100 µL of the erythrocyte solution and incubation for one hour at 37 °C. Subsequently, the total content was transferred to the tubes. Finally, after centrifugation at 1500 rpm for 5 min, 100 µL of the supernatant was removed, transferred to another microplate, and shaken at 1000 rpm for 1 min to remove air bubbles. The optical density was measured at a wavelength of 450 nm. The percentage of hemolysis was calculated using the following equation:

$$\%\;hemolysis=\frac{\left(EA\;absorbance-PBS\;absorbance\right)\times 100}{\left(triton\;absorbance-PBS\;absorbance\right)}$$

### 4.9. Cytotoxicity Assay

The cytotoxic activities of EA, FLZ, and AE + FLZ were evaluated in human cervical carcinoma cells (HeLa, ATCC^®^ CCL-2™). Cells were seeded in 96-well microplates at a density of 2.5 × 10^4^ cells per well and supplied with growth medium consisting of Dulbecco’s Modified Eagle’s medium (DMEM) with 10% fetal bovine serum (FBS), 100 UI/mL penicillin, and 100 μg/mL streptomycin. The cells were incubated at 37 °C in a 5% CO_2_ atmosphere. A series of concentrations of the tested agents, EA (62.5–4000 μg/mL), FLZ (16–256 μg/mL), AE + FLZ (31.25 + 0.5 to 500 + 8 μg/mL), and vehicle (78–1250 µg/mL NaOH), were added to each well of the plate and incubated for 24, 48, and 72 h. Afterwards, the cells were washed, 10 μL of MTT dye in a 5 mg/mL solution was added to each well, and the plate was incubated in CO_2_ at 37 °C for 4 h. Next, the cells were dissolved in ethanol and the optical densities were measured at 570 nm. Negative controls were obtained by measuring the untreated cell culture medium. Data from the control and treated cells were calculated and expressed as the percentage of viable cells [55].

### 4.10. Statistical Analysis

The test results were subjected to the Shapiro–Wilk normality test (except for MIC, CFM, and in vitro interaction). Significant differences between groups were determined by one-way analysis of variance (ANOVA), followed by Tukey’s multiple comparison test when the sample was considered normally distributed, and Kruskal–Wallis analysis followed by Dunn’s test for abnormal distribution in GraphPad Prism^®^ 9.0, where the value *p* < 0.05 was considered statistically significant. All experiments were performed in triplicate in three independent experiments.

## 5. Conclusions

The results of our study are promising, indicating that combining EA and FLZ can potentially reduce the amount needed to suppress or eliminate *C. albicans* growth in vitro. This may result in lower doses and possibly diminished cytotoxic effects. However, it is crucial to investigate the mechanisms underlying the antifungal activity of EA, its interaction with FLZ, and its efficacy in animal studies. Current research emphasizes the difficulties in using EA therapeutically in vivo, mainly because of its limited solubility in water and consequent poor bioavailability. Additional studies are needed to improve EA’s water solubility and bioavailability. These research efforts aim to develop EA, both alone and in combination with FLZ, as an effective antifungal treatment, particularly for infections caused by *C. albicans*.

## Figures and Tables

**Table 2:** In vitro activity of the combination of ellagic acid with fluconazole against *C. albicans*.

Microorganisms	MIC (μg/mL)		FIC (μg/mL)		FICI	Interaction
	EA	FLZ	EA	FLZ		
Reference strains						
*C. albicans* (ATCC 90028)	500	2	125	1	0.75	Additive
*C. albicans* SC5314 (ATCC MYA-2876)	250	8	250	0.5	1.06	Indifferent
Clinical isolates						
*C. albicans* CA 02	500	8	250	0.5	0.56	Additive
*C. albicans* CA 04	500	16	250	2	0.62	Additive
*C. albicans* CA 05	1000	32	250	1	0.28	Synergistic
*C. albicans* CA 08	1000	64	62.5	2	0.09	Synergistic
*C. albicans* CA 010	1000	64	125	2	0.15	Synergistic
*C. albicans* CA 013	1000	64	125	2	0.15	Synergistic
*C. albicans* CA 014	250	32	250	2	1.06	Indifferent
*C. albicans* CA 015	500	64	62.5	2	0.15	Synergistic
*C. albicans* CA 016	500	64	62.5	4	0.18	Synergistic

Legend: ellagic acid (EA), fluconazole (FLZ), Minimum Inhibitory Concentration (MIC), fractional inhibitory concentration (FIC), Fractional Inhibitory Combination Index (FICI): synergism: ≤0.5; additive: 0.5 < FICI ≤ 1; indifference: 1 < FICI ≤ 4; and antagonism: FICI > 4 [24]. All experiments were performed in triplicates in three independent experiments.

**Table 3:** Total indices of ellagic acid + fluconazole combinations obtained in the checkerboard test.

Strains	Fractional Inhibitory Combination Index (FICI)	
	Synergistic	Additive
CA 02	-	0.56, 0.62, 0.75, 1.0
CA 04	-	0.62, 0.75, 1.0
CA 05	0.28, 0.31, 0.37, 0.5	0.53, 0.56, 0.62, 0.75, 1.0
CA 08	0.09, 0.12, 0.15, 0.18, 0.25, 0.28, 0.31, 0.37, 0.5	0.53, 0.56, 0.62, 0.75, 1.0
CA 010	0.15, 0.18, 0.25, 0.28, 0.31, 0.37, 0.50	0.53, 0.56, 0.62, 0.75, 1.0
CA 013	0.15, 0.18, 0.25, 0.28, 0.31, 0.37, 0.50	0.53, 0.56, 0.62, 0.75, 1.0
CA 015	0.15, 0.18, 0.25, 0.28, 0.31, 0.37	0.53, 0.56, 0.62, 0.75, 1.0
CA 016	0.15, 0.18, 0.25,0.28, 0.31, 0.37, 0.5	0.53, 0.56, 0.62, 0.75, 1.0

-: No interaction; FICI (Fractional Inhibitory Combination Index): synergism: ≤0.5; additive: 0.5 < FICI ≤ 1; indifference: 1 < FICI ≤ 4.0; and antagonism: FICI > 4.0 [24].

**Table 4:** Identification and origin of *C. albicans* isolates.

Microorganisms	Origin
Strains ATCC 90028 and SC5314 ATCC MYA-2876	ATCC
Clinical isolates CA 01, CA 03, CA 04, CA 05, CA 06, CA 09, *CA* 010, CA 011, CA 013, CA 014, CA 016, and CA 017	Urine
CA 02, CA 07, CA 012, CA 015, CA 019, CA 020, and CA 021	Tracheal secretion
CA 08	Sputum
CA 018	Blood culture

ATCC: American Type Culture Collection; clinical isolates were obtained from different patients.

## Data Availability

The original contributions presented in this study are included in the article. Further inquiries can be directed to the corresponding author.
